# Polythiophenes Comprising Conjugated Pendants for Polymer Solar Cells: A Review

**DOI:** 10.3390/ma7042411

**Published:** 2014-03-27

**Authors:** Hsing-Ju Wang, Chih-Ping Chen, Ru-Jong Jeng

**Affiliations:** 1 Institute of Polymer Science and Engineering, National Taiwan University, Taipei 106, Taiwan; E-Mail: allisonwang101@gmail.com; 2 Department of Materials Engineering, Ming Chi University of Technology, 84 Gunjuan Road, Taishan, New Taipei City 243, Taiwan; 3 Battery Research center of Green Energy, Ming Chi University of Technology, 84 Gunjuan Road, Taishan, New Taipei City 243, Taiwan

**Keywords:** polythiophene, polymer solar cells, conjugated pendants, energy level engineering

## Abstract

Polythiophene (PT) is one of the widely used donor materials for solution-processable polymer solar cells (PSCs). Much progress in PT-based PSCs can be attributed to the design of novel PTs exhibiting intense and broad visible absorption with high charge carrier mobility to increase short-circuit current density (*J*_sc_), along with low-lying highest occupied molecular orbital (HOMO) levels to achieve large open circuit voltage (*V*_oc_) values. A promising strategy to tailor the photophysical properties and energy levels via covalently attaching electron donor and acceptor pendants on PTs backbone has attracted much attention recently. The geometry, electron-donating capacity, and composition of conjugated pendants are supposed to be the crucial factors in adjusting the conformation, energy levels, and photovoltaic performance of PTs. This review will go over the most recent approaches that enable researchers to obtain in-depth information in the development of PTs comprising conjugated pendants for PSCs.

## 1. Introduction

Polymer solar cells (PSCs) comprising conjugated polymer donor and fullerene derivative acceptor materials are a promising alternative for producing clean and renewable energy [1]. This is because of the potential to fabricate large areas of lightweight flexible substrates by solution processing at a low cost. The power conversion efficiency (PCE) of PSC has been steadily increased from *ca*. 1% in 1995 quickly to ~9% recently for single junction devices and ~11% for tandem devices with fairly good stability [2–7]. Several significant milestones in PSC researches have been established on the way to the realization of practical applications.

**Figure 1.**
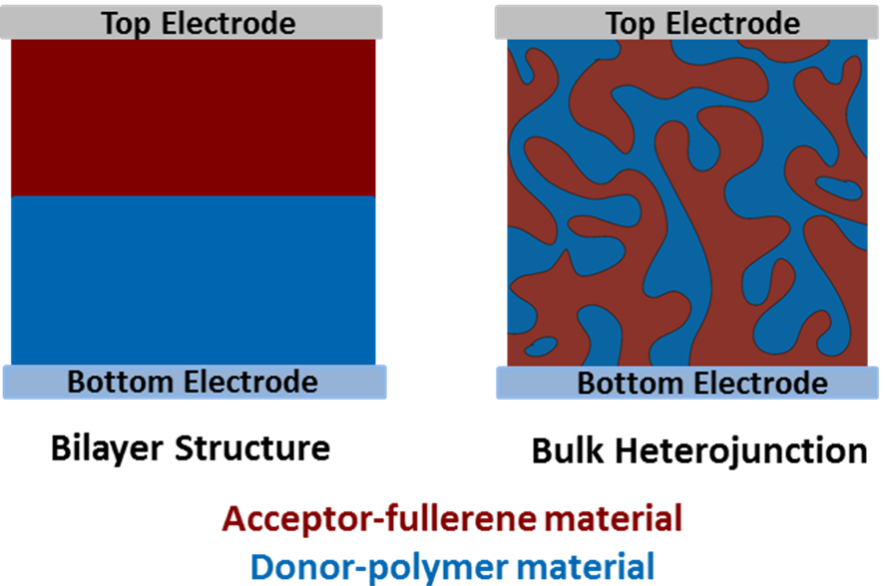
Fundamental architectures of photoactive layer: bilayer anf bulk heterojunction.

Two fundamental architectures of photoactive layer, namely bilayer structure and bulk heterojunction (BHJ) structure, have been developed for solar cells (Figure 1). The bilayer heterojunction solar cell was initially reported by Tang in 1986, and the device is formed by separately depositing donor and acceptor materials, resulting in two layers with a sharp, well-defined interface [8]. A solution-processed BHJ is the most successful device architecture for organic photovoltaics because exciton harvesting is entirely by creating a highly folded architecture such that all excitons are formed near a heterojunction interface. There has been intense research on developing novel organic electron donor (p-type) and electron acceptor (n-type) materials, including polymer/fullerene, small molecule/fullerene, and all-polymer systems, for solution processed BHJ photovoltaic devices during the last decade [4–6]. An interpenetrating network of BHJ with a large “donor-acceptor” (D/A) interfacial area enabled the minimization of excitons traveling distance (electron-hole pair generated upon light absorption) to the D/A interface, thereby ensuring the exciton dissociation at the D/A interface to generate maximum free charge carriers. Additionally, through controlling the nanoscale phase-separated domain morphology between the D/A components in BHJ, charge transport (CT) pathways are provided to facilitate the charge transfer state separating into mobile electrons and holes. After charge separation at the D/A interface, holes and electrons need to travel to positive and negative electrodes through donor and acceptor networks, respectively. Subsequently, corresponding anode and cathode would collect the charges to complete the conversion from the photon energy to electrical energy (*i.e*., photovoltaic effect).

**Figure 2.**
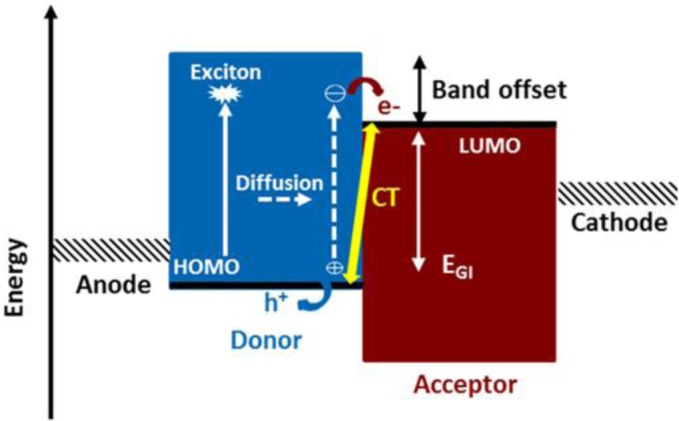
Basic geometry and energy level diagram of the BHJ cell.

The basic geometry and the illustration of energy level diagram for polymeric BHJ solar cells are illustrated in Figure 2. When a light irradiates the photoactive layer through the transparent electrode side, the photoactive layer would absorb photons in the range of its absorption band to generate excitons (bound electron-hole pairs). The exciton subsequently undergoes diffusion until it reaches the BHJ (D/A) interface. This is known as the charge transfer state, and mobile free electrons and holes are formed from this state. However, this electron transfer of exciton does not necessarily produce free (dissociate) charge carriers in a direct manner. In general, the electron-hole pair located on photoactive materials is expected to exhibit a significant Coulomb attractive force. Figure 2 also illustrates the energy bands and the interface optical transitions as compared to the bulk transitions. Due to the energy loss associated with the band offset to overcome this Coulomb attraction, the exciton can dissociate at the interface [9–11]. In principle, the *E*_g_ of p-type material is considered as the sum of *E*_GI_ and band offset in order to discuss the energy band configuration of BHJ. The energy difference between the HOMO level of p-type material and the lowest unoccupied molecular orbital (LUMO) level of n-type material is defined as *E*_GI_. A larger value of E_GI_ certainly leads to a higher *V*_oc_. On the other hand, the LUMO-LUMO offset between p/n-type materials refers to be band offset as illustrated in Figure 2. The adequate band offset must be large enough to separate the tightly bound excitons in an effective manner. In other words, the band offset provides a driving force to split the excitons. However, the band offset should not be too large in order to maintain a sufficient *E*_GI_, *i.e*., to approach a satisfactory *V*_oc_. [12]. Finally, the separated electrons and holes then will transport along D/A interpenetrating network toward the metal cathode and anode, respectively, and are to be collected by the electrodes to form photocurrent and photovoltage. The information about the electronic structure of BHJ solar cells points out that the energy-level alignment plays a crucial role on determining the performance of a photovoltaic cell.

**Figure 3.**
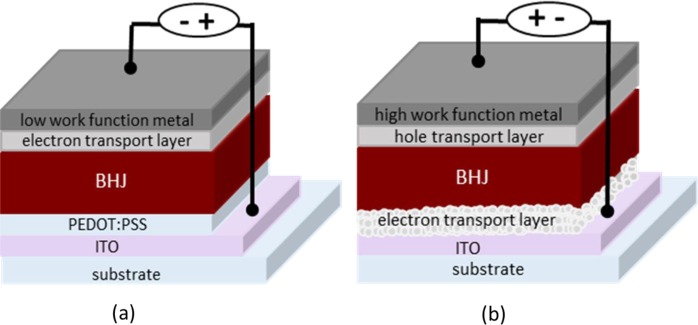
Device architecture of (**a**) conventional and (**b**) inverted PSCs.

Two main classifications of device geometries are currently in use including the conventional and the inverted types schematically represented in Figure 3 [13,14]. In the conventional geometry (Figure 3a), the layer stack is usually built on top of a semitransparent indium tin oxide (ITO) electrode followed by a hole transport layer poly(3,4-ethylenedioxythiophene):poly(styrene sulfonate) (PEDOT:PSS), an active layer, an electron transport layer (e.g., lithium fluoride or calcium) and finally, a low work function metal electrode (the cathode). In the inverted type geometry (Figure 3b), the order of the layers is reversed with the top metal electrode now being the hole-collecting anode. TiO_2_, ZnO are the most widely studied electron transport materials to be inserted between ITO and the active layer of inverted PSCs [15–17]. It is important to note that different types of metal electrodes can be used in these two cases. Low work function metals such as aluminum and/or calcium are typically used as back electrodes in the conventional cells while higher work function metals such as silver are used in the inverted cells. Different orders of the layers in the two geometries create interfaces with various surface interactions, which may affect the photovoltaic performances. Interestingly, the inverted geometry devices tend to be much more stable than the conventional one, which are intended as durable devices in the view point of life-time issue [18].

**Figure 4.**
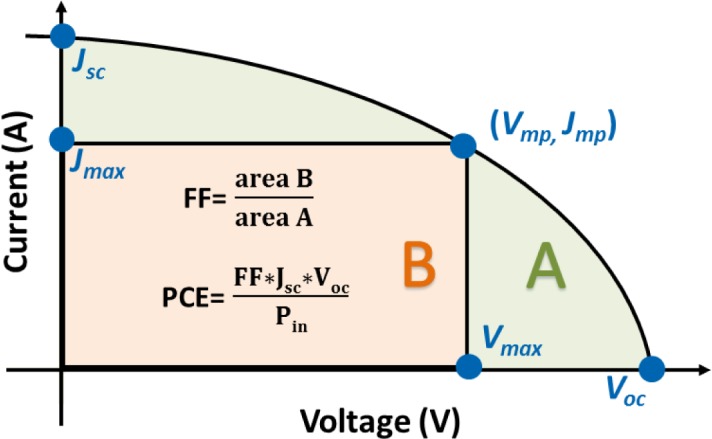
Typical current density (*J*)-voltage (*V*) curve of PSCs.

A typical current density (*J*)-voltage (*V*) curve of a PSC device is shown in Figure 4. The PCE of a PSC is proportional to short circuit current (*J*_sc_), open circuit voltage (*V*_oc_), and fill factor (FF). *J*_sc_ depends on the efficiencies of the light absorption of the active layer, exciton diffusion to and dissociation at the D/A interface, charge transportation in the active layer, and charge collection on the electrodes. *V*_oc_ is mainly proportional to the energy level difference (*E*_GI_ in Figure 2) between the LUMO of the acceptor and the HOMO of the donor. Lower HOMO levels of the polymers would provide a higher *V*_oc_ [19]. Additionally, conjugated polymers with higher ionization potentials are capable of minimizing the *p*-doping level under ambient O_2,_ and hence increasing the environmental stability.

Several reviews have surveyed the account of novel conjugated polymers for solar cell applications [20–24]. Among many promising conjugated polymers for solar cells, polythiophene (PT)-based materials exhibit an irreplaceable position due to their unique conductivity and optical properties [25–31]. The control of the HOMO-LUMO energy levels (*i.e*., band gaps) of PT-based materials has been in the center of the synthetic chemistry of functional π-conjugated systems. Herein, in the viewpoint of photovoltaic applications, a profound assay on the chemistry of PT systems toward piecing together new molecular constitutions with the corresponding optical and electronic properties has been set forth.

**Figure 5.**
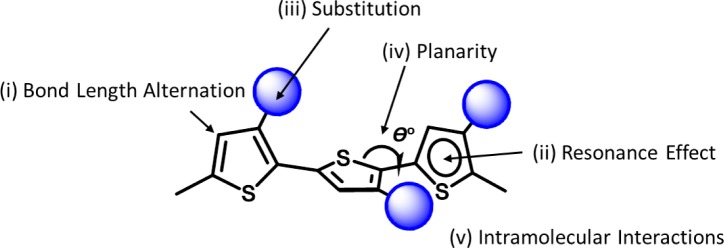
Structural factors determining the band gap of PTs.

The band gap of PT can be expressed by the sum of five contributions, as shown in Figure 5, which are (i) bond length alternation; (ii) resonance effect; (iii) introduction of electron-deficient or electron-sufficient substituent; (iv) dihedral angle θ between consecutive units; and (v) intermolecular interactions [32]. For these factors, the absorption properties and the energy level can be modulated by introducing conjugated substituents into the side chains and main chains of PTs. Among a big family of PTs, poly(3-hexylthiophene) (P3HT) is the most commonly used material due to many advantages such as easy synthesis, high charge carrier mobility, and good processability. Unfortunately, the main issue with P3HT is its large optical band gap (*E*_g_^opt^) (~1.9 eV) and high HOMO level, which lead to insufficient absorption in visible region, and the poor *V*_oc_ value of the fabricated devices. To address the energy level mismatch and insufficient absorption issues of PTs, several synthetic strategies have been developed and proven to be very effective: (i) to construct the backbone using alternating thiophene (electron-rich donor) and strong electron withdrawing substitute (electron-deficient) units to form D-π-A PTs; (ii) to synthesize a rigid fused planar ring to stabilize the quinoid resonance structure; (iii) to attach conjugated side chains on the polymer main chains. The details can be found in recent reviews on the chemistry of the materials [21–23].

Among numerous approaches, a series of side chain conjugated polymers have attracted considerable research interests [33]. Side chains in conjugated polymers have been primarily developed as the solubilizing groups. However, these side chains have roles that are far beyond the improvement of solubility of studied polymers. In this review, we especially promote using side chain engineering to tune the physical properties of PTs, including absorption, emission, energy level, molecular packing, and charge transport. The tailoring photophysical properties and energy levels via covalently attaching electron donor and acceptor pendants on PTs will be further addressed in this article.

Firstly, we will introduce the development history and the chemistry of PTs. Subsequently, we will highlight some recent examples using side chain engineering to manipulate the photophysical and electrical performance. The category encompasses side chains with functional moieties directly connected to the conjugated backbones may be divided in two items: electron-donating and electron-withdrawing groups.

As mentioned in previous section, many PTs have been built and explored over the past decades. A breakthrough in new materials with high efficiency single junction PSC device is summarized as the following. Yu *et al*. [34,35] first reported the synthesis of the fused thieno[3,4-b]thiophene (TT) building block, which can stabilize the quinoid structure to reduce the bandgap. Yang *et al*. [36] reported the synthesis of the benzo[1,2-b;4,5-b]dithio-phene (BDT) unit for PSCs (Scheme I). This large planar BDT unit has emerged as an attractive building block for high-efficiency photovoltaic polymers. Simultaneously, Chen *et al*. [37,38] also synthesized a thiophene-based ladder type copolymer composed of indacenodithiophene (IDT) unit for PSCs and a PCE of 6.4% was obtained (Scheme II). Based on the success of BDT and IDT units incorporated on conjugated polymer structure for PSC applications, it is reasonable to figure out that conjugated side chain engineering can also be carried out using the BDT and IDT units. In the last section, we will especially introduce a section that BDT and IDT containing polymers set the milestones of high PCEs among various p-type polymer candidates by utilizing side chain engineering approach.

## 2. The Developement History of Polythiophene

PTs are the most important classification of conjugated donor polymers utilized in a broad area of applications such as conducting polymers [37], light-emitting diodes [38], field-effect transistors [39], and polymer solar cells due to the excellent optical and electrical properties. The structure of a PT with regioregular conformation is shown in Scheme III. To improve the solubility of PTs, many different alkyl-substitutes have been explored. Given the fact that the thiophene ring is a 5-membered ring, the thiophene monomer is polymerized through the 2- and 5-position substitution, introducing the directionality in the polymer. Whenever a monomer is incorporated in the growing polymer chain, the monomer can be added with the head (2-position) or the tail (5-position) first. However, a mixture of the possible couplings usually results in a regiorandom poly(3-alkylthiophene) in which a large number of thiophene rings twist out of conjugation planarity in a manner of head to head coupling due to steric repulsion between alkyl chains, as illustrated in Scheme IV. A random sequence distribution reduces the electrical conductivity of PTs. With coupling each thiophene unit in a consecutive head-to-tail manner during the polymerization, one is able to afford a regioregular poly(3-alkylthiophene) which is capable of adopting a coplanar conformation, resulting in a lower energy. Highly regioregular poly(3-hexylthiophene) (*rr*-P3HT) can be obtained via McCullough [40], Rieke [41] or Grignard metathesis (GRIM) [42] methods. The enhancement of regioregularity in P3HT through these advanced metal-catalyzed reactions leads to various beneficial outcomes including a red shift in absorption in the solid state, an intensified extinction coefficient, and an increase in the mobility of the charge carriers. Since the advent of this regioregular concept, numerous groups have been trying to enhance the photovoltaic performances by modifying the blend morphology of *rr*-P3HT:PCBM in nanoscale. Indeed, the morphology of the photoactive polymer-fullerene blend can be affected by controlling several production parameters during the film formation or by treatments afterwards including thermal-annealing, solvent-annealing, replacing solvent, and so on [43–49]. However, the main issue with P3HT is its narrow absorption range in visible region and low oxidation potential, which lead to insufficient visible region photon absorption, low *V*_oc_ values, and electrochemically unstable of the corresponding devices.

Attaching conjugated side chains onto PTs differs strategically from the previous linearly conjugated PT cases. To achieve better absorption in visible region and lower HOMO levels, the tailoring of photophysical properties and energy levels via covalently attaching electron donor and acceptor pendants on PTs has attracted much attention. Considering that the polymerization usually takes place on 2- and 5-position of the thiophene ring, there are many routes for incorporating the conjugated pendants on thiophenes. Similar to the construction of polymer backbone during polymerization, the efficient C–C single bond formation between main chain and conjugated pendants lies essentially on the cross coupling between two unsaturated carbons in the aromatic units. Usually the reaction involves a transition-metal-catalyzed oxidative addition reaction across the C–X bond of an electrophile and then trans-metalation with a main group organometallic nucleophile, followed by a reductive elimination step leading to the C–C bond formation. Famous reactions used on such direct attaching via single bond are Kumada-Coupling [50], Stille-Coupling [51], and Suzuki-Miyaura Coupling [52]. In addition, the syntheses of vinylene-containing conjugated linkers via the carbon−carbon double bond formation between two respective monomers are Wittig-Horner reaction, Horner-Wadsworth-Emmons reaction and Knoevenagel condensation [53–56]. The palladium-catalyzed C–C coupling between aryl halides (or vinyl halides) and activated alkenes in the presence of a base is referred as Heck Reaction [57]. Functionalized alkyene containing conjugated pendants could be prepared by Sonogashira-Coupling, with the usage of a palladium catalyst to form a carbon–carbon bond between a terminal alkyne and an aryl or vinyl halide [58].

## 3. Side Chain Engineering of PT Derivatives

Effective light harvesting is crucially important for PSC applications. PTs functionalized with conjugated pendant groups possess broad absorption band in the UV and visible regions, and consequently can harvest a greater amount of solar light, which is advantageous for photovoltaic applications. In 2006, Li *et al*. [59] first designed and synthesized a series of PT derivatives having conjugated bi(thienylenevinylene) side chains (Scheme Va, P3-1-1, P3-1-2, P3-1-3) via Stille coupling. It has been shown that the extension of a conjugated side chain to the PT main chain leads to strong and broad absorption covering both the UV and visible regions from 350 to 650 nm. In this particular work, a series of PTs exhibit two absorption peaks located in the UV and visible regions. The UV peak results from the absorption of the bi(thienylenevinylene) side chains (350–450 nm), and the visible peak is attributed to the absorption of the π–π* transition of the conjugated polymer main chains (450–650 nm). In addition, *E*_g_^opt^ of PTs (1.98 eV in average) are similar to that of *rr*-P3HT, but their HOMO and LUMO levels positively shifted by *ca.* 0.2 eV in comparison with those of P3HT. The solar cell device fabricated with P3-1-3/PC_61_BM (w/w, 1:1) reached a PCE of 3.18%. When compared with P3HT-based devices using similar fabricated condition, an obviously improvement of about 38% was demonstrated for the P3-1-3-based PSC sample.

Not only intramolecular charge transport (ICT) along the conjugated main chains, but also intermolecular charge hopping between adjacent main chains is the controlling step in the charge-transport process. In addition to increasing molecular weight or controlling regioregularity of conjugated polymers to induce higher hole mobility, side chains comprising crosslinkable bridges would enhance conjugation, and π–π stacking interactions between PT main chains. A series of PTs cross-linked by different amounts of conjugated vinylene-terthiophene-vinylene bridges (**PT-VTThV2**, **PT-VTThV4**, and **PT-VTThV8**), were synthesized via Stille coupling (Scheme Vb) [60]. The crosslinked bridges connected and conjugated with the PT backbone would provide efficient charge transport through electron communication between chain-to-chain networks. However, the crosslinking of the conjugated bridges distorted the polymer main packing and hence decreased the degree of planarity to some extent, resulting in the blue shift of the absorption and the *E*_g_^opt^ enhancement (2.29 eV for **PT-VTThV2**, 2.31 eV for **PT-VTThV4**, and 2.37 eV for **PT-VTThV8**). The hole mobilities of **PT-VTThV2**, **PT-VTThV4**, and **PT-VTThV8** are 4.7 × 10^−3^, 2.58 × 10^−3^, and 9.48 × 10^−4^ cm^2^·V^−1^·s^−1^, respectively, according to a space-charge-limited current (SCLC) model. With a 2% cross-linking content, the hole mobility of **PT-VTThV2** is about a thousand times higher than that of the corresponding non-bridge-conjugated polymer (5.23 × 10^−6^ cm^2^·V^−1^·s^−1^ for **P1**). However, one needs to balance the trade-off between adequate cross-linking content and polymer backbone planarity. In case of **PT-VTThV4** and **PT-VTThV8**, the steric hindrance between molecules and distortion in the main chain were inflected for decreasing hole mobility and blue-shifting absorption spectra. Finally, a PCE of 1.72% for the solar cell device based on a **PT-VTThV2**/PC_61_BM (w/w, 1:1) blend film was obtained.

Bilge *et al*. [61] developed a series of swivel-cruciform oligothiophene dimers for solution-processed organic field-effect transistors (OFET). Afterward, they studied the influence of such 3,3′-bithiophene conjugated bridge (Monomer 1, Scheme VIa) within a P3HT backbone on solid state interchain packing, hole mobility and photovoltaic properties in PSCs [62]. The conjugated bridges were introduced via a random copolymerization approach with a McCullough-type GRIM (**B-P3HT**). Increasing the degree of conjugated bridge (Monomer 1) from 2%, 4%, to 8% in the P3HT backbone would suppress the degree of solid-state ordering and the formation of ordered P3HT domains. This further resulted in a blue-shifted absorption, a lower hole mobility and a decreased performance of PSC, despite the improvement of solubility. An optimized PCE of about 2% and a hole mobility of about 3.1 × 10^−4^ (cm^2^·V^−1^·s^−1^) were derived from the solar cell based on the B-P3HT comprising 2% of conjugated bridges blended with PC_61_BM (1:1, w/w). However, the presence of an excessive amount of conjugated bridges (**B-P3HT 8%**) resulted in sacrificing the hole mobility (6.4 × 10^−6^ cm^2^·V^−1^·s^−1^) and an unsatisfactory PCE of 0.13%.

In an attempt to obtain broad absorption and desirable hole mobility, Chen *et al*. [63] developed regioregular PTs possessing alkyl-thiophene side chains. A series of regioregular side chain decorated PTs (**P3-4-1** and **P3-4-2**) were obtained by Stille coupling reaction (Scheme VIb). To avoid conformational disorder induced by *cis*/*trans* conformations of the vinylene bonds, the alkyl-thiophenes were directly attached onto the PT backbones without vinylene linkers. The X-ray crystallography revealed a nearly planar conformation of side chain monomer. The longer conjugated side chains improved the degree of electronic communication and lower *E*_g_^opt^ (1.98 eV for **P3-4-1** and 1.77 eV for **P3-4-2**). This led to high hole mobility was obtained by organic thin film transistor measurement (3.5 × 10^−4^ cm^2^·V^−1^·s^−1^ for **P3-4-1** and 4.6 × 10^−3^ cm^2^·V^−1^·s^−1^ for **P3-4-2**). Moreover, low-lying HOMO levels of **P3-4-1** and **P3-4-2** (−5.46 eV and −5.62 eV) were observed from electrochemical studies. As a result, a positive and impressive contribution to a higher *V*_oc_ value of 0.91 V of **P3-4-1** based PSCs was observed. Unfortunately, **P3-4-2** exhibited poor solubility in *o*-DCB and resulted in undesirable PSC performance. A PCE of 2.5% was achieved for the **P3-4-1**/PC_71_BM based PSC under white light illumination (AM 1.5G, 100 mW/cm^−2^).

According to the theory of mesogen-jacketed liquid crystalline polymers: when side chains are attached laterally to the gravity centers of the main chain without spacers, the main chain of polymers are forced to be extend and to be conformed rigidly because of high population of both bulky and rigid side groups around the backbone and the “jacket” is formed [64,65]. Following the design concept, ordered mesogen arrangement offers a great opportunity to elongate the conjugation via chain alignment through incorporating rigid side chains into the π-conjugated polymers. Kuo *et al*. [66] synthesized three different kinds of side chain type PT derivatives employing mesogen-jacketed-like design (Scheme VII). These side chain type PTs were synthesized by attaching terthiophene groups to the 3-position of the thiophene rings in the polymer backbone via a vinyl linkage with different spatial arrangements (**P3-5-1**, **P3-5-2**, and **P3-5-3**). To systematically examine the geometrical effect of the pendants on the optical, physical, and electrochemical properties of the side chain type PTs, these terthiophenes pendants are linked as perpendicular or parallel position via vinyl group with respect to the PT main chains. The film of **P3-5-3** revealed the most red-shifted absorbance profile in the wavelength range between 450 and 700 nm. HOMO energy levels of the three polymers were in the following order: **P3-5-1** (−4.98 eV) > **P3-5-2** (−5.25 eV) > **P3-5-3** (−5.31 eV). Featureless XRD diffraction pattern of **P3-5-1** implies that the chain-packing into ordered lamella structure was prohibited due to the oversized side chains. On the other hand, the pendants incorporated in parallel to the main chains (**P3-5-2** and **P3-5-3**) of PTs would assist the elongation of π-conjugation aided by the terthiophene jacket structure, as evidenced by the presence of distinct diffraction peaks. The side-chain orientation impacted photophysical and packing morphology was evaluated. The structure-property relationship in conjugated side-chain PTs offers inspiration for the design of promising photovoltaic materials.

## 4. PTs with Electron Donor Conjugated Side Chains

Electron-donating side chains can contribute some degree of electron density to the conjugated polymer backbones through connecting moieties. The increasing hole mobility of polymer can be obtained with the incorporation of electron donating pendants. Triphenylamine (TPA) is a preferred electron-donating moiety with excellent hole transporting properties. In fact, TPA derivatives have been widely investigated for almost two decades [67,68]. For instance, soluble regioregular TPA-containing PT derivatives were synthesized by Grignard metathesis method (Scheme VIIIa) [69]. Because of the solubility issue, the dialkoxy-substituted TPA moiety was designed and incorporated during GRIM polymerization. The intramolecular energy transfer was found from the dialkoxy-substituted TPA moieties to the PT backbones. In comparison with the TPA-based PTs without dialkoxy substitution in dilute solution, broader peaks in the visible absorption were pronounced for the PTs polymers possessing dialkoxy-substituted TPA pendants. Electrochemical characterizations revealed that PTs containing TPA moieties as side chains showed relatively higher oxidation potential and lower HOMO energy level when compared to P3HT. This is beneficial to the enhancement of air-stability and *V*_oc_ values. The *V*_oc_ values of the PSC samples using the blends of these materials as donor and PC_61_BM as acceptor (w/w = 1:1) reached 0.71–0.87 V along with the PCEs in the range of 0.014% and 0.45% under white light at 100 mW/cm^2^.

Wang *et al*. [70] synthesized low band-gap PT derivatives, with bulky conjugated side-chains composed of TPA, thiophene, and vinylene groups (TPATh) (Scheme VIIIb). These PTs, synthesized by GRIM and Stille coupling with different copolymer configurations and side chain densities, are regioregular-TPATh-PT (***rr*-TPATh-PT**) and random-TPATh-PT (***r-*TPATh-PT**), respectively. It was found that the steric hindrance of bulky conjugated moiety curtails the effective conjugation length in the main chain. Because of this, low HOMO levels were obtained for the copolymers (−5.38 eV for ***rr*-TPATh-PT** and −5.35eV for ***r*-TPATh-PT**). Moreover, ***r*-TPATh-PT** with less bulky side-chain content exhibited better conjugation along the polymer backbone than did ***rr*-TPATh-PT**. Higher absorption intensity in the visible region was observed for ***r*-TPATh-PT** (100,158 cm^−1^ at λ_max_ *≈* 434 nm) in comparison with ***rr*-TPATh-PT** (86,856 cm^−1^ at λ_max_ *≈* 421 nm). The hole-transporting property was strongly dependent on the side-chain density of the PT derivatives. The PT derivative with a lower side chain density would provide sufficient free volume and consequently resulted in better compatibility for the ***r*-TPATh-PT**/PC_61_BM-blend film as compared to the ***rr*-TPATh-PT**/PC_61_BM-blend film. Better PCE performances of the ***r-*TPATh**-PT/PC_61_BM-based PSCs (0.94%) were observed in comparison with the ***rr*-TPATh**-PT/PC_61_BM-based PSCs (0.49%). Further improvement of PSC performance was achieved for the PSC sample fabricated from the blend of ***r-*TPATh-PT**/PC_71_BM (w/w = 1:3), with a *J*_sc_ of 6.83 mA·cm^−2^, a *V*_oc_ of 0.71 V and a PCE of 1.75%.

Zhang *et al*. [71] designed a series of PTs with styryl–triphenylamine (TPA) as side chains along with unsubstituted tetrathienyl spacers (**PT5TTPA**, Scheme VIIIc). When compared to previously reported PTs comprising bulky pendants, the side chain isolated-PT exhibited red-shifted and enhanced π–π* transition absorption of the polymer backbone along with the shoulder peak and steep absorption edge, indicating improved planarity of the polymer backbone. The solution and film absorption of **PT5TTPA** displayed similar broad absorption bands from 300 to 650 nm with three distinct absorption peaks: the first belongs to the π–π* transition of TPA group at 303 nm, the second is attributed to the absorption of the thiophene units with the conjugated side chain at 413 nm, and the last is the maximum absorption of the copolymer backbone at 555 nm. The unsubstituted thiophene spacer along the polymer backbone of the side chain isolated-PTs presented a lower HOMO energy level at −5.1 eV. The polymer solar cell based on **PT5TTPA** as donor and Indene-C60 bisadduct (ICBA) as acceptor revealed a PCE of 3.6% with a high *V*_oc_ of 0.94 V, under the illumination of AM1.5G, 100 mW/cm^2^. The results indicate that the strategy for broadening absorption by the attachment of isolated bulky side chains is efficient for improving the PCE of PSCs.

Wang *et al*. [72] synthesized several PTs, namely **PTBPTPA**, **PTStTPA**, and **PTCNStTPA** (Scheme IX), featuring three different conjugated units–biphenyl (BP), stilbene (St), and cyanostilbene (CNSt), respectively, in the PT backbones, and conjugated triphenylamine/thiophene (TPATh) moieties as side chain. In addition, three conjugated BP-, St-, and CNSt-based main-chain-type conjugated polymers (**PTBP**, **PTSt**, and **PTCNSt**, respectively) were also synthesized. However, poor solubility of co-monomer CzPh would bring about the resulting CzPh**-PT** copolymer with a unsatisfactory weight average molecular weight (lower than 10 kg/mole). Moreover, the HOMO energy levels of the BP-, St-, and CNSt-based main-chain-type (−5.61 to −5.41 eV) and side–chain–type (−5.37 to −5.16 eV) PTs were lower than that of P3HT. Because of this, a high *V*_oc_ of 0.86 V was achieved by the **PTStTPA**/PC_61_BM-based (w/w = 1:1) PSC device. Moreover, the photovoltaic performances of PSCs fabricated from blends of the side-chain-type PTs and the fullerene derivative PC_61_BM were superior to those of the PSCs based on the main-chain-type polymer/PC_61_BM blends.

Carbazole (Cz) is also a promising candidate as electron donor moiety for optoelectronic materials [73,74] With that in mind, Wang *et al*. prepared a PT copolymer (***r*-CzPh-PT**) containing 9-position substituted carbazole-phenyl (CzPh) as bulky pendants (Scheme Xa) [75]. Electrochemical study presented that the onset oxidation potential positively shifted 0.47 eV for ***r*-CzPh-PT** in comparison with that of P3HT. By incorporating the 9-position substituted carbazoles as bulky pendant groups increases the steric hindrance between molecules of ***r*-CzPh-PT**, the effective conjugation length could be curtailed, and consequently the lowered HOMO energy level (−5.38 eV) was acquired. This led to better air stability and a high *V*_oc_ value for the PSC sample. However, the poor solubility of copolymerized monomer of CzPh led to the ***r*-CzPh-PT** copolymer with unsatisfactory weight average molecular weight (lower than 10 kg/mol). This would certainly have a negative impact on the film quality and nanoscale morphology. The PSC device based on the blend of ***r*-CzPh-PT**/PC_61_BM (w/w = 1:1) gave an overall PCE of only 0.36% with a *V*_oc_ of 0.85 V, a *J*_sc_ of 1.57 mA/cm^2^, and a FF of 0.27. Poor PCE performance was because of the presence of coarse phase separation in the ***r*-CzPh-PT**/PCBM blend film as investigated by atomic force microscopy (AFM).

Due to the steric hindrance of the bulky conjugated moiety, the conjugated pendant-containing thiophene monomer shows low reactivity and poor solubility. PTs with relatively lower molecular weights will be derived when such monomers are polymerized. Wang *et al*. [76] further synthesized novel conjugated triphenylamine- or carbazole-containing pendants substituted with *tert*-butyl groups (*t*TPA and *t*Cz, respectively) to overcome solubility issue. In an attempt to stabilize the morphology and miscibility between the polymer/fullerene blend films, the *tert*-butyl groups was decorated on pendants owing to the better solubility in organic solvents. Thus, a series of PTs featuring 2-ethylhexyl-substituted terthiophene (T) or quaterthiophene (BT) as the conjugated unit in the polymer backbone with pendant conjugated *t*TPA or *t*Cz as side chains, namely **PT*t*TPA**, **PBT*t*TPA**, **PT*t*Cz**, and **PBT*t*Cz** (Scheme Xb), were polymerized for PSCs. The strategy of incorporating T and BT moieties onto the polymer backbone along with appending *t*TPA or *t*Cz units as the side chains were developed to facilitate ICT within extended conjugated frameworks of the polymers, resulting in not only lower band-gap energies of PTs, but red-shift of the maximal UV-Vis absorption wavelengths as well. The presence of better electron-donating *t*TPA resulted in broader absorption bands and lower *E*_g_^opt^ of **PT*t*TPA** (1.65 eV) and **PBT*t*TPA** (1.58 eV) as compared with **PT*t*Cz** (1.80 eV) and **PBT*t*Cz** (1.77 eV). In addition, better compatibility between the polymer and PC_61_BM was observed for the BT-containing PTs than for the T-containing PTs. It was found that **PBT*t*TPA/**PC_61_BM and **PBT*t*Cz/**PC_61_BM blend films based PSCs showed superior photovoltaic performance to **PT*t*TPA** and **PT*t*Cz** composite films. The PCE of 2.77% was achieved by the **PBT*t*TPA**/PC_61_BM blend film (w/w = 1:3) based PSC sample.

## 5. PTs with Electron Acceptor Conjugated Side Chains

Another approach to manipulate the energy levels of PT is the introduction of an electron acceptor group as the pendant on polymer backbone. Chochos *et al*. [77] have synthesized novel cyano containing *n*-type PT derivatives (P3CN4HT and PBCN4HT, Scheme XIa), in which the cyano group is attached to the 4-position of the repeat unit in *rr*-P3HT. By the addition of copper cyanide (CuCN) to P3B4HT (Scheme XIa) along with using hexamethylphosphortriamide (HMPA) as polar solvent at a reflux temperature of *ca.* 190 °C, P3CN4HT was successfully synthesized. This polymer exhibited high electron affinity for n-type semiconductor and excellent solubility in common organic solvents. When compared to the UV-Vis spectrum of the P3B4HT (λ_max_ = 338 nm) in solution, the relatively red-shifted absorption maxima of PBCN4HT (λ_max_ = 369 nm) and P3CN4HT (λ_max_ = 392 nm) were observed due to the fact that the cyano group is more planar and less bulky than the bromine atom. This probably allows more efficient packing of the polymer chains. According to electrochemical study, the values for the HOMO and the LUMO energy levels were calculated at −6.1 eV and −3.6 eV for P3CN4HT, respectively, implying the potential for *n*-type material applications. When P3CN4HT was employed for the blends as the electron acceptor, the completely photoluminescence quenching for both poly[2-methoxy-5-(3′,7′-dimethyloctyloxy)-1,4-phenylenevinylene] (MDMO-PPV):P3CN4HT and poly3-octylthiophene (P3OT):P3CN4HT mixtures were observed. A PCE of 0.014% for the MDMO-PPV:P3CN4HT (w/w = 1:2) blend film was obtained via preliminary photovoltaic measurements.

Wei and co-workers reported a series of regioregular 3-hexylthiophene copolymers incorporated with different composition ratios of octylphenanthrenyl-imidazole moieties by GRIM polymerization (Scheme XIb) [78]. With 90 mol% octylphenanthrenyl-imidazole moieties onto P3HT chains, the *E*_g_^opt^ was effectively reduced from 1.91 eV to 1.80 eV as compare to the parent P3HT. In addition, charge separation was also facilitated through sequential transfer of electrons from the main chains to the side chains, and then to PC_61_BM. Consequently, the PSC device based on a copolymer presenting 90 mol% octylphenanthrenyl-imidazole moieties as donor material blended with PC_61_BM as acceptor (w/w = 1:1) exhibited an overall PCE of 3.45% along with a *J*_sc_ of 13.7 mA/cm^2^, a *V*_oc_ of 0.68 V, and a FF of 37.2%.

As mentioned earlier, the polymers presenting bulky conjugated electron donor moieties as side-chains reveal that the sterically induced twisting of the polymer backbone is responsible for achieving a low-lying HOMO level, and enhancing the oxidative stability of conjugated polymers as compared to the parent P3HT. However, the bulky substituents increase the degree of twisting from planarity in the backbone, resulting in a decreased ICT and larger *E*_g_^opt^. Alternating donor-acceptor units on PTs reveal a lower *E*_g_ due to the presence of the efficient ICT. That inspires researchers to systematically investigate the variation of photophysical properties and energy levels via the strategy of developing PTs covalently attached with both electron donor and acceptor pendants, in other words, the ICT from the PT backbone to the conjugated acceptor side chain. Wang *et al*. [79] first synthesized PTs functionalized with various contents of *t*Cz as electron donor pendant, and bisbenzothiazolylvinyl (DBT) as electron acceptor pendant (Scheme XII). Due to the electron withdrawing characteristic of DBT moiety accompanied by the stronger donor-π-acceptor affect and more efficient ICT, the red-shift of the maximum absorption wavelengths for the PTs with increasing DBT content were taken place. The energy level engineering of PTs was heavily influenced by the geometry steric hindrance of bulky conjugated pendants capable of twisting adjacent thiophene planar, thereby reducing the π orbital overlapping of PTs backbones and resulting in the deeper HOMO energy levels to some extent (−5.26 to −5.39 eV). Notably, this phenomenon was more pronounced in the *t*Cz-rich PTs. By adopting the blends of each PTs and PC_71_BM to prepare conventional PSC devices, high *V*_oc_ values of around 0.79–0.91 V were achieved. The PCE of the PSCs based on **PT*t*Cz**:PC_71_BM (w/w = 1:2.5) reached 2.48% with a *V*_oc_ of 0.91 V, *J*_sc_ of 6.58 (mA·cm^−2^) and FF of 41% under the illumination of AM1.5, 100 mW/cm^−2^. To further investigate the performance of the low-lying HOMO material in the study of long-term stability, an inverted PSC with **PT*t*Cz**/PC_71_BM sandwiched between ZnO*_x_* (as an electron extraction layer) and MoO_3_ (a hole extraction layer). This inverted PSC was capable of retaining *ca.* 80% of its original efficiency after storage under ambient conditions (without encapsulation) over 1000 h, according to the ISOS-D-1 shelf protocol.

To avoid the distortions in the conjugated backbone induced by bulky withdrawing substituents, fluorine atom substituents are of interest on account of their size complementarity with hydrogen. The van der Waals radius is 1.35 Å for fluorine atom and 1.10 Å for hydrogen atom. Moreover, fluorine with drastically different electronic properties from those of hydrogen, exhibits Pauling electronegativity of 4.0, ready to be another candidate as smallest electron-withdrawing group. Gohier *et al*. [80] prepared 3-fluoro-4-hexylthiophene by a synthetic route involving perbromination of 3-hexylthiophene followed by the protection of 2- and 5-positions of thiophene by trimethylsilyl groups and bromine/fluorine exchange (**3T-H**, **3T-F**, and **3T-Br** in Scheme XIII). Poly(**3T-H**), poly(**3T-Br**), and poly(**3T-F**) were obtained by electropolymerization of terthienyls **3T-H**, **3T-F**, and **3T-Br**. The measurement of optical properties of the polymers utilized the samples prepared from the direct deposition on indium tin oxide (ITO) electrodes, and revealed band gap energies, such as 1.99 eV for poly(**3T-H**), 1.89 eV for poly(**3T-Br**), and 1.87 eV for poly(**3T-F**). The substitution hydrogen by halogen atoms led to a slight decrease of the band gap (*ca.* 0.10 eV). The electron-withdrawing effect of the halogen atoms stabilizes the HOMO energy level and, thus, leads to an increase of the oxidation potentials of the prepared polymers. In comparison of two halogen atoms: Br and F exhibited similar effects on the electronic properties of the conjugated system. However, a slightly lower energy gap for the fluorinated compounds and a slightly higher oxidation potential for the brominated compounds were observed. This study sheds the light on the introduction of fluorine atoms onto PTs to increase oxidation potential and thus to help the stability toward oxidative degradation.

## 6. Side Chain Engineering of Benzodithiophene (BDT)-Based Conjugated Polymers

In 2008, the bandgaps, molecular energy levels, and photovoltaic performances of BDT-based polymers with commonly used conjugated units (thiophene, benzothiadiazole (BT), thienopyrazine (TPZ), *etc*.) had first been investigated systematically by Yang’s group [36]. At the same time, BDT has been widely chosen for donor unit because of its deep HOMO energy level of the resulting polymer, structural symmetry of the rigid fused aromatic system to enhance the electron delocalization, and interchain interaction to improve the charge mobility by Yu’s group [81–83]. Beyond the linear low energy level engineering, two dimensional conjugated system using BDT as the building block attracted much more attention. In 2010, Huo *et al*. [84] further incorporated additional thiophene units as pendants to the BDT-based conjugated backbones (**PBDTTBT**, Scheme XIVa). **PBDTTBT** exhibited three absorption bands in the range 300–700 nm both in chloroform and as solid film. The main absorption peak of **PBDTTBT** was located at approximately 581 nm in solution and red-shifted to 596 nm as solid film with a stronger shoulder at 627 nm. The *E*_g_^opt^ of **PBDTTBT** as solid film was 1.75 eV. In addition, the HOMO (−5.31 eV) and LUMO (−3.44 eV) energy levels of **PBDTTBT** were calculated from electrochemical studies. A PCE of 5.66% was obtained from the **PBDTTBT** derived device, with a *V*_oc_ of 0.92 V, a *J*_sc_ of 10.7 mA·cm^−2^, and a FF of 57.5%. The high *V*_oc_ value of **PBDTTBT**-based device resulted from its low-lying HOMO level. Huo *et al*. [85] also designed a 5-alkylthiophene-2-yl-substituted BDT monomer with thieno[3,4-b]thiophene (TT) as conjugated unit, and synthesized **PBDTTT**-based polymers with alkoxycarbonyl group or alkylcarbonyl groups on the TT unit. Afterward, they further synthesized two new **PBDTTT**-based polymers possessing either the thienyl-substituted BDT with alkoxycarbonyl-substituted thieno[3,4-b]thiophene (TT-E) or the alkylcarbonyl-substituted thieno[3,4-b]thiophene (TT-C), namely **PBDTTT-E-T** and **PBDTTT-C-T** (Scheme XIVb) [86]. The main absorption bands of **PBDTTT-E-T** and **PBDTTT-C-T** were clearly red-shifted and broadened to some extent when compared to those of their analogous polymers without alkylthienyl substituents. Again, the enhanced intermolecular π–π interaction of the two-dimensional (2D) conjugated polymers was demonstrated by the effectively extended conjugation of alkylthienyl side chains. The PSCs based on the 2D conjugated polymers clearly exhibited better photovoltaic performances than the PSCs based on their analogous polymers without alkylthienyl substituents. Notably, the enhancement of *J*_sc_ values of the PSCs based on **PBDTTT-E-T** and **PBDTTT-C-T** was significant due to the increased hole mobility and the broadened absorption of the 2D conjugated structure. Overall, the PSC sample based on **PBDTTT-C-T** exhibited an impressive PCE of 7.59%.

A new conjugated copolymer **PBDTTT-TIPS** containing thiazolothiazole acceptor unit and triisopropylsilylethynyl (TIPS)-functionalized BDT donor unit was synthesized via Stille polymerization (Scheme XIVc) [87]. TIPS moiety in essence is a kind of acene derivatives with many advantages including the improvement of solubility, the stabilization of the oxidative potential of the semiconductors, and the enhancement of π-orbital overlap. As a result, solution-processed PSCs based on the blend of **PBDTTT-TIPS** and PC_71_BM exhibited a PCE of 4.33% without any post-treatments. When compared to long-branch alkyl-substituted polymers, the presence of TIPS side chains promotes nano-scale interpenetration morphology with ideal domain sizes around 10–20 nm in the TIPS-containing polymer/PC_71_BM blend. This is beneficial to charge separation and enhanced efficiency.

Zhang *et al*. [88] synthesized a series of 2D-conjugated D-π-A based on BDT or benzodifuran (BDF) as the donor unit, BT as the acceptor unit, and thiophene as the π-bridge for the application as p-type materials in PSCs (Scheme XVa). The polymers based on BDT with thiophenes or furans as side chains were named as **PBDTT-BT** and **PBDTF-BT**, respectively. Meanwhile, **PBDFT-BT** based on BDF with a thiophene as side chain and **PBDFF-BT** based on BDF with a furan as side chain were synthesized for investigating the differences of five-membered sulfur or oxygen heterocyclic ring compounds on the photovoltaic properties of the copolymers. The absorption edges of the four polymer films were located at 739 nm for **PBDTT-BT**, 728 nm for **PBDTF-BT**, 738 nm for **PBDFT-BT**, and 771 nm for **PBDFF-BT**, corresponding to the *E*_g_^opt^ values of 1.67, 1.70, 1.68 and 1.61 eV respectively. In addition, the HOMO levels of **PBDTT-BT**, **PBDTF-BT**, **PBDFT-BT** and **PBDFF-BT** are −5.26, −5.24, −5.08 and −5.11 eV, respectively. Apparently, the HOMO energy levels of the copolymers based on BDF are *ca*. 0.1 eV up-shifted in comparison with that of the polymers based on BDT. This is because the diameter of the oxygen atom is smaller than that of the sulfur atom. Therefore, the BDF unit possesses an even better planar structure and consequently a better conjugated backbone than that of the copolymers based on the BDT unit. For the same reason, higher mobilities of BDF based polymers were achieved in comparison with those of the copolymers based on the BDT unit. The hole mobilities are 2.5 × 10^−5^, 6.1 × 10^−4^, 9.0 × 10^−3^ and 3.0 × 10^−4^ cm^2^·V^−1^·s^−1^, for **PBDTT-BT**, **PBDTF-BT**, **PBDFT-BT**, and **PBDFF-BT**, respectively. The PCEs of the PSCs based on the polymers as the donor and PC_71_BM as the acceptor with a 1:1 donor–acceptor weight ratio reached 1.85% for **PBDTT-BT**, 2.88% for **PBDTF-BT**, 4.42% for **PBDFT-BT**, and 2.60% for **PBDFF-BT**, respectively. Obviously, the PSC sample derived from **PBDFT-BT** (based on the **BDF** unit and thiophene conjugated side chains) exhibited the best PCE in this study.

In order to achieve a low-lying HOMO energy level of fluorinated BDT-based polymer, Son *et al*. [89] described the synthesis of fluorinated polythienothiophene(TT)-*co*-BDT (Scheme XVb) and the characterization of their physical properties, especially in the aspect of solar cells. The results indicate that the attachment of fluorine to the polymer backbone did not change its optical properties significantly. A similar absorption profile was obtained. Nevertheless, the fluorination of the polymer backbone exerted a significant effect on lowering the HOMO levels of polymers (−4.94 eV for **PTBF0**, −5.11 eV for **PTBF1**, −5.41 eV for **PTBF2**, and −5.48 eV for **PTBF3)**. The fluorinated backbones would also introduce a driving force for phase separation of the polymers with PC71BM when blended together. The photovoltaic properties of the polymers were studied with the structure of ITO/PEDOT:PSS/polymer:PC_71_BM/Ca/Al. The active layers were spin-coated from the solutions of the polymer and PC_71_BM (in o-DCB), and the optimized weight ratios of polymer to PC_71_BM were 1:1, 1:1.5, 1:1.5, and 1:1.5 for **PTBF0**, **PTBF1**, **PTBF2**, and **PTBF3**, respectively. Indeed, the devices based on the fluorinated polymers exhibited the enhancement of *V*_oc_ as compared with the devices based on the non-fluorinated polymer (0.58 V for **PTBF0**, 0.74 V for **PTBF1**, 0.68 V for **PTBF2**, and 0.75 V for **PTBF3**). This is because of the differences in the HOMO energy levels. Moreover, the **PTBF2** and **PTBF3** polymers were photochemically much more unstable as compared to **PTBF0** and **PTBF1** under ambient atmosphere. Further investigation on the photochemical stabilities of polymers demonstrated that the fluorination of polymer provides an internal polarization to stabilize polymers against singlet oxygen attack.

Zhou *et al*. [90] reported a highly robust polymer**-PBnDT-DTffBT** (Scheme XVIa), which is composed of a widely used strong electron deficient unit 5,6-difluoro-4,7-dithien-2-yl-2,1,3-benzothiadiazole (DTffBT) and donor unit benzo[1,2-*b*:4,5-*b*′]dithiophene (BnDT). A polymer **PBnDT-DTBT** composed of non-fluorine containing acceptor unit DTBT was synthesized for comparison. The absorption maximum of **PBnDT-DTffBT** in chlorobenzene solution was red-shifted by approximately 80 nm when the measuring temperature dropped from 100 °C to room temperature. This implies that fluorine atoms exerted a great influence on inter- and intramolecular interactions through C–F···H, F···S, and C–F···πF interactions among the fluorinated polymer chains. This led to the aggregation of the polymer at a rather low temperature in solution state. Additional absorption shoulder was revealed for **PBnDT-DTffBT** as thin film. This is possibly due to the polymer-chain stacking in the solid state. To study the fluorination effect on the energy levels, the HOMO/LUMO level presented a down-shifted from −5.40/−3.13 eV for **PBnDT-DTBT** to −5.54/−3.33 eV for **PBnDT-DffTB**. The lower HOMO level of **PBnDT-DTffBT** would bring about a larger *V*_oc_ of 0.91 V for the **PBnDT-DTffBT**-based PSC device. A BHJ device derived from **PBnDT-DffTB** demonstrated a high PCE of 7.2%. A similar strategy by incorporating BDT as the donor and either benzotriazole (HTAZ) or its fluorinated analogue (FTAZ) as the acceptor was also reported (Scheme XVIb) [91]. A PCE of 7.1% along with a *V*_oc_ of 0.79 V, a *J*_sc_ of 12.45 mA/cm^2^, and an impressive *FF* of 72.2% were achieved by the PSC sample based on this fluorinated polymer **PBnDT−FTAZ** blended with PC_61_BM. The promising PCE of BDT containing 2D conjugated polymers for PSCs are summarized in Table 1.

## 7. Side Chain Engineering of Indacenodithiophene (IDT)-Based Conjugated Polymers

A family of IDT-based conjugated polymers has also attracted attention as a donor unit in the D-A systems for photovoltaic applications in recent years. This is because the coplanarity of the IDT unit could enhance interchain interaction of the polymers and lead to higher hole mobility [92–94]. Chen *et al*. [95]. first synthesized a series of copolymers based on IDT with aryl substituents (Scheme XVIIa, **PTPTBT**) **PTPTBT** possessed lower-lying HOMO energy levels at −5.36 eV and a PCE of 6.1% was achieved for the PSC sample based on this particular copolymer. Using side chain energy level engineering strategy, Chen *et al*. [96] further synthesized a **PIDT-FQ** conjugated polymer (Scheme XVIIb), with IDT as the donor unit and di-fluorophenylquinoxaline (FQ) as the acceptor unit. **PIDT-FQ** presented a desirable HOMO level at −5.36 eV along with an *E*_g_^opt^ of 1.81 eV. The PSCs based on **PIDT-FQ/**PC_71_BM with solvent annealing process showed a PCE of 4.6% with a high *V*_oc_ of 0.90 V. Subsequently, Chen *et al*. [97] synthesized an IDT-based donor conjugated polymer (**PIDTHT-BT**), with conjugated *n*-hexylthiophenes as side chains (Scheme XVIIc). By extending the degree of intramolecular repulsion through the side chain engineering strategy, a HOMO energy level of −5.46 eV was obtained. This enabled a fabricated solar cell to exhibit a high *V*_oc_ of about 0.9 V. The hole mobility in a **PIDTHT-BT**/PC_71_BM (w:w = 1:4) blend film is 2.2 × 10^−9^ m^2^·V^−1^·s^−1^. The **PIDTHT-BT**/PC_71_BM (w:w = 1:4) PSC exhibited a satisfactory PCE of 4.5% with a *J*_sc_ of 9.1 mA/cm^2^, a high *V*_oc_ of 0.9 V, and a *FF* of 52.7%. The promising PCE of BDT containing 2D conjugated polymers for PSCs are summarized in Table 2.

## 8. Conclusions and Perspectives

This review emphasizes on the role of conjugated PTs comprising conjugated pendants in the development of high performance conjugated polymers for PSCs. Polymers with such features are capable of tuning PSC-relevant characteristics including absorption, emission, energy level, molecular packing, and charge transport. These are key factors that one should consider in the aspect of selecting robust pendant groups. Apparently, the prospect of PSCs would mainly count on further ingenious approaches in developing novel high performance conjugated PTs comprising conjugated pendants.

The following requirements in the molecular design of novel polymers comprising conjugated pendants should be kept in mind: (1) Conjugated pendant-containing monomers exhibit low reactivity and poor solubility due to steric hindrance. Hence, the incorporation of the alkyl groups on conjugated pendants would impart better solubility along with improved thin film processability and higher molecular weights of the synthesized polymers; (2) Adequate side chain density is a must to guarantee better main chain planarity, whereas and the diminishment of bulky side chain steric hindrance could strengthen π–π stacking and increase hole mobility; (3) In attempt to enhance the molecular packing and charge transport of 2D conjugated system, rigid fused unit (BDT or IDT)-containing copolymers possess planar structures with high hole mobility and suitable electronic energy levels; (4) The electron-withdrawing fluorine substitution on the acceptor units of the polymers results in lower HOMO level of the polymers, and higher *V*_oc_ and PCE of the PSCs, which is an effective way to improve the photovoltaic performance.

The choice of conjugated pendants attached onto the PT framework certainly plays an important part in tailoring the photovoltaic performance of PSCs. Generally, conjugated units as the functional pendants of PTs are greatly beneficial to the enhancement of *J*_sc_ values. This is because such structure design would bring about broadening absorption and higher hole mobility. Further incorporation of electron donor or acceptor pendants serves to manipulate HOMO or LUMO level to an optimum. This would lead to large *V*_oc_ values. In addition, the geometrical and steric structures of conjugated pendants are most likely to interfere the molecular packing of polymer backbone and nanoscale phase separation of the interpenetrating BHJ network. This could be favorable for achieving better PSC performance. Apart from that, D-A copolymers composed of planar units with conjugated side chains as donor, and fluorinated substituents as acceptor seem to be a good candidate for acquiring large *J*_sc_ and *V*_oc_. Undoubtedly, in pursuit of high performance PSCs for practical applications, PTs functionalized with robust conjugate side chains exhibit highly promising prospect.

## Figures and Tables

**Scheme I.**
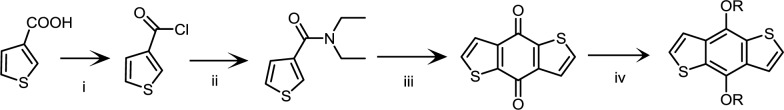
Synthesis of the BDT unit: (i) oxalyl chloride, methylene chloride, ambient temperature, overnight; (ii) diethylamine, methylene chloride, ambient temperature, 30 min; (iii) *n*-butyllithium, THF, ambient temperature, 30 min; then water, several hours; (iv) Zn, NaOH, H_2_O, reflux for 1 h; then *n*-C*_n_*H_2_*_n_*_+1_Br, TBAB, reflux for 6 h.

**Scheme II.**
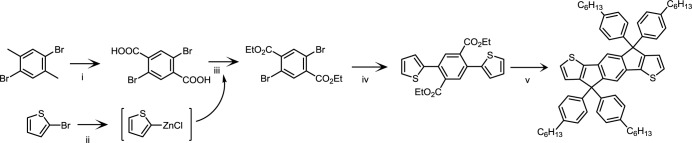
Synthesis of the IDT unit: (i) KMnO_4_/H_2_O, pyridine, reflux; (ii) Mg, I_2_, THF for 1 h; then ZnCl_2_ at 0 °C for 3 h; (iii) EtOH, H_2_SO_4_, reflux, 6 h; (iv) Pd(PPh_3_)_4,_ THF, 12 h; (v) *p*-tolylmagnesium bromide, ether for 2 h; then HOAc, HCl reflux for 12 h.

**Scheme III.**
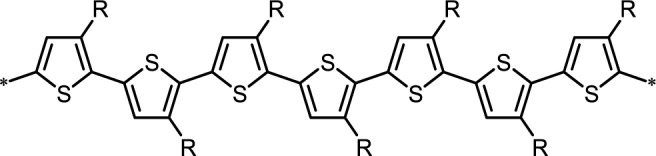
Poly(3-alkylthiophene) with regioregular conformation.

**Scheme IV.**
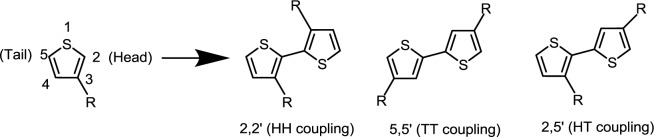
Possible couplings of 3-alkylthiophenes.

**Scheme V.**
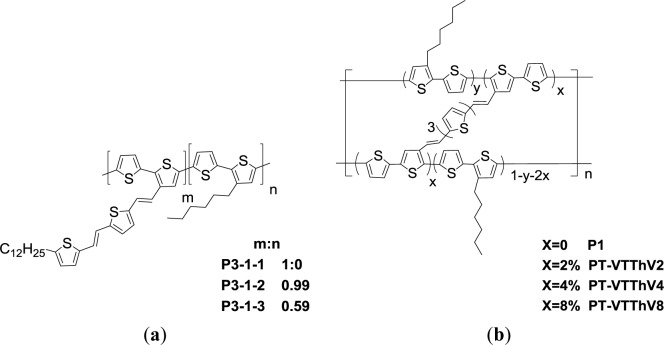
Chemical structures of (**a**) PTs with conjugated bi(thienylenevinylene) side chains and (**b**) PTs cross-linked by conjugated vinylene-terthiophene-vinylene bridges.

**Scheme VI.**
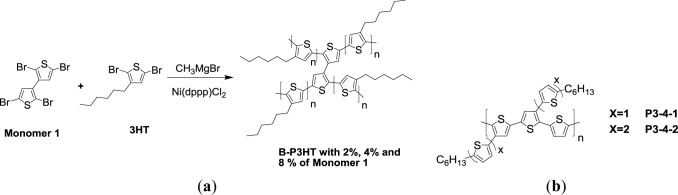
(**a**) Synthesis of P3HT with 3,3′-bithiophene conjugated bridge and (**b**) chemical structure of regioregular PTs possessing alkyl-thiophene side chains.

**Scheme VII.**
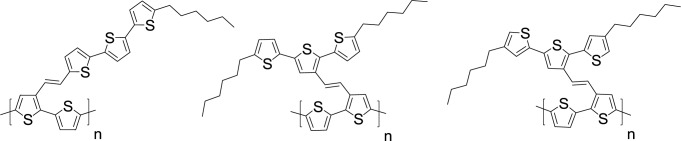
Chemical structures of PTs employing mesogen-jacketed-like design.

**Scheme VIII.**
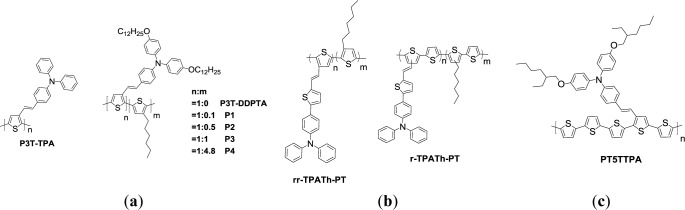
Chemical structures of TPA-containing PTs.

**Scheme IX.**
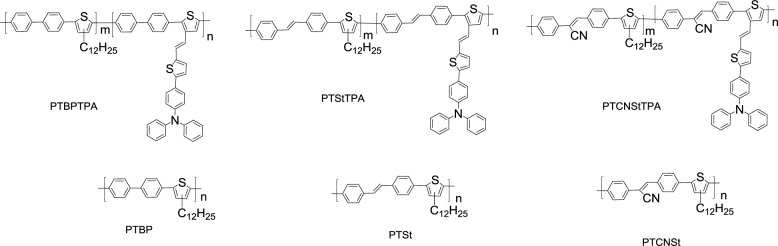
Chemical structures of side-chain- and main-chain-type PTs.

**Scheme X.**
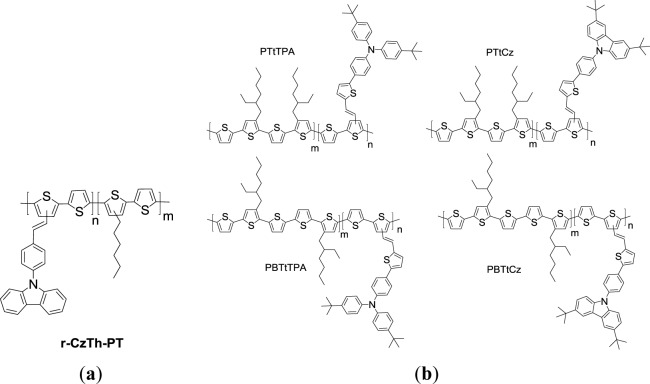
Chemical structures of (**a**) *r-*CzPh-PT and (**b**) PTs with TPA or Cz pendants.

**Scheme XI.**
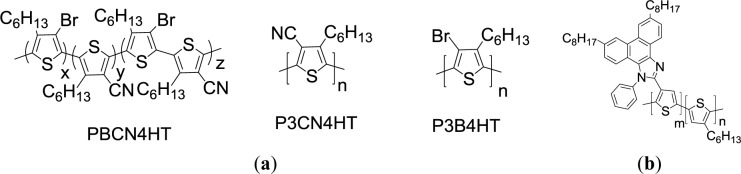
Chemical structures of (**a**) P3CN4HT, PBCN4HT, P3B4HT and (**b**) PTs with octylphenanthrenyl-imidazole moieties.

**Scheme XII.**
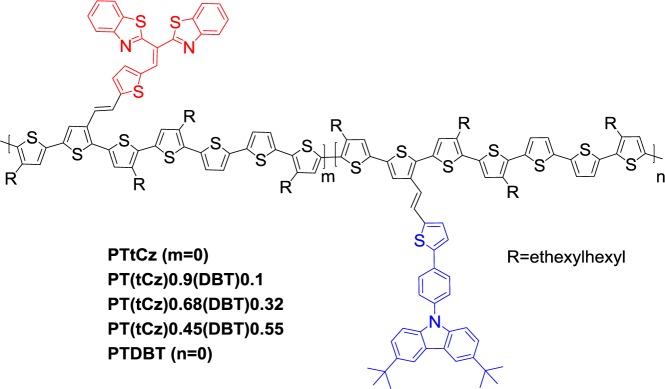
Chemical structures of PTs with *t*Cz and DBT pendants.

**Scheme XIII.**
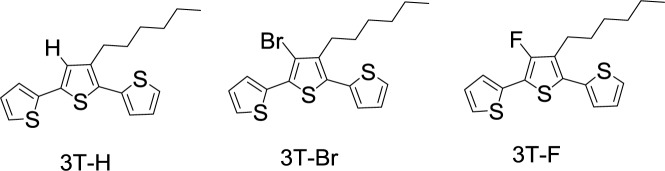
Chemical structures of 3T-H; 3T-Br; and 3T-F.

**Scheme XIV.**
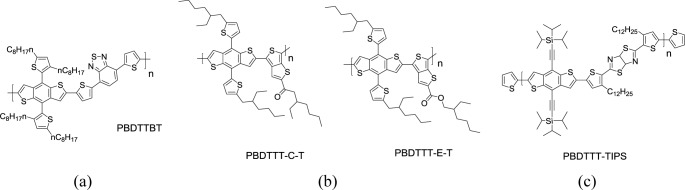
Chemical structures of (**a**) PBDTTBT; (**b**) PBDTTTs; and (**c**) PBDTTT-TIPS.

**Scheme XV.**
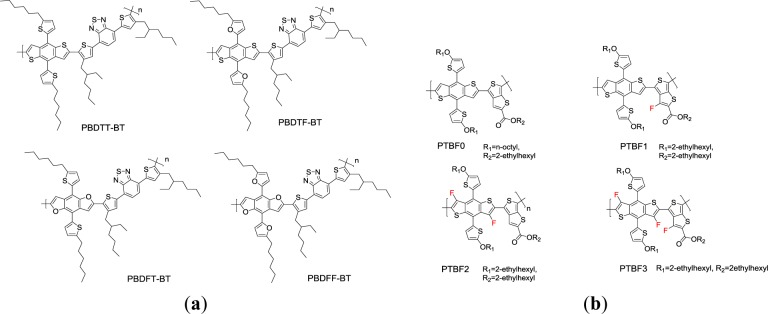
Chemical structures of (**a**) BDT/BDF based PTs and (**b**) fluorinated BDT-based PTs.

**Scheme XVI.**
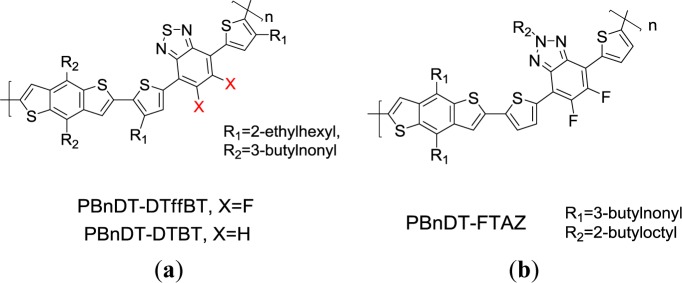
Chemical structures of (**a**) PBnDT-DTffBT, PBnDT-DTBT and (**b**) PBnDT−FTAZ.

**Scheme XVII.**
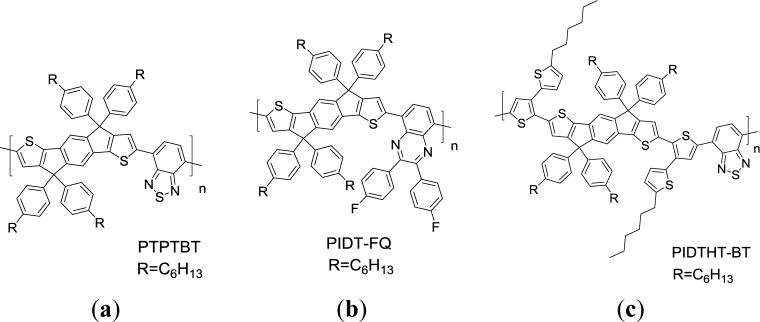
Chemical structures of IDT-based 2D conjugated PTs.

**Table 1.** BDT-based 2D conjugated polymers for PSC.

Polymer	Fullerene	p:n (w:w)	*J*_sc_ (mA/cm^2^)	*V*_oc_ (V)	FF (%)	PCE (%)	Reference
PBDTTBT	PC_71_BM	1:2	10.7	0.92	57.5	5.66	[84]
PBDTTT-C-T	PC_71_BM	1:1.5	17.48	0.74	58.7	7.59	[86]
PBDTTT-TIPS	PC_71_BM	1:1	9.77	0.89	49.8	4.33	[87]
PBDTF-BT	PC_71_BM	1:1	9.94	0.73	60.9	4.22	[88]
PTBF1	PC_71_BM	1:1.5	14.1	0.74	68.9	7.2	[89]
PBDT-DTHBT	PC_61_BM	1:1	12.91	0.91	61.2	7.2	[90]
PBDT-FTAZ	PC_61_BM	1:2	12.45	0.79	72.2	7.1	[91]

**Table 2.** IDT-based 2D conjugated polymers for PSC.

Polymer	Fullerene	p:n (w:w)	*J*_sc_ (mA/cm^2^)	*V*_oc_ (V)	FF (%)	PCE (%)	Reference
PTPTBT	PC_71_BM	1:2.5	11.2	0.85	67.2	6.4	[95]
PIDT-FQ	PC_71_BM	1:2.5	9.2	0.90	55.6	4.6	[96]
PIDTHT-BT	PC_71_BM	1:4	9.1	0.93	52.7	4.6	[97]
